# Redistribution of China’s Green Credit Policy among Environment-Friendly Manufacturing Firms of Various Sizes: Do Banks Value Small and Medium-Sized Enterprises?

**DOI:** 10.3390/ijerph18010033

**Published:** 2020-12-23

**Authors:** Yuming Zhang, Chao Xing, David Tripe

**Affiliations:** 1 School of Management, Shandong University, Jinan 250100, China; zhangym@sdu.edu.cn; 2 School of Economics and Finance, Massey University, Palmerston North 4442, New Zealand; D.W.Tripe@massey.ac.nz

**Keywords:** green credit, environment-friendly manufacturing enterprises, small and medium-sized enterprises, banks loans, China

## Abstract

According to previous studies, China’s green credit policy, which was launched in 2012, increases environment-friendly manufacturing enterprises’ loan amounts. In this paper, we focus on a redistribution mechanism among environment-friendly manufacturing firms, namely, we determine whether the effects of the green credit policy differ between small and medium-sized environment-friendly manufacturing enterprises (SMEMEs) and large environment-friendly manufacturing enterprises (LEMEs). Using a difference in difference model (DID) and a difference in difference in difference model (DDD), we find that SMEMEs obtain more loans than LEMEs due to the green credit policy. We further analyze three potential foundations of this redistribution mechanism: information asymmetry, financial development, and government environmental investment. The results demonstrate that the redistribution effect occurs in both low and high information asymmetry conditions but only in regions with satisfactory financial development and with lower government environmental investment. Our findings enrich the literature on green credit, sustainable finance, and small finance, and they provide references for enterprises, banks, and governments.

## 1. Introduction

In recent years, with the deterioration of the natural environment, calls for considering both economic development and environmental protection have been rapidly increasing. In practice, environment-friendly manufacturing enterprises, which refer to firms that are embedded in the business field of environmental protection products, e.g., clean energy production, water conservation equipment, and recycling products, are the main force for realizing environmental protection via commercial methods [1]; hence, the development and performance of these firms is vital to the economy and the environment [2]. In this context, China, which is one of the largest emerging markets and most polluting countries, has implemented a green credit policy to financially support environment-friendly manufacturing enterprises.

China’s green credit policy was launched by the China Banking Regulatory Commission and other central government departments in 2012. It requires commercial banks to increase loan support for the green economy, especially by investing more funds in eco-friendly enterprises and projects, and it prohibits these banks from extending loans to any polluting firm or product [3,4]. Due to the “carrots and sticks” nature of the green credit policy, China’s environment-friendly manufacturing enterprises and the firms with superior environmental performance can obtain better loan contracts after its implementation, while loan finance for heavily polluting enterprises has become more difficult [5,6]. Therefore, the green credit policy establishes a “distribution effect”, namely, the provision of more loans to environment-friendly manufacturing enterprises.

However, the loans may not be equally distributed among environment-friendly manufacturing enterprises that differ in terms of size. Since China’s ecological industry is in the initial phase, with small and medium-sized environment-friendly manufacturing enterprises (SMEMEs) accounting for a larger proportion of environment-friendly manufacturing enterprises, the impact of the green credit policy on them is uncertain [7]. SMEMEs can realize higher environmental performance due to their efficient and flexible operation [8,9], which is valued by the green credit policy. Nevertheless, the long-term problems of information asymmetry and the liability of small size contribute to a mismatch of financial resources [10], and in combination with the high uncertainty and failure risks of the ecological market [11,12], SMEMEs’ access to banks loans may still be constrained. Thus, in this paper, we explore several important questions: How do banks evaluate SMEMEs in the context of China’s green credit policy? Do banks value the efficient operating ability of SMEMEs and increase loan amounts? Or do they only extend large loans to large environment-friendly manufacturing enterprises (LEMEs) due to the disadvantages of SMEMEs? In other words, What type of redistribution effect does the green credit policy have on environment-friendly manufacturing enterprises with various corporate scales?

There is a readily identifiable gap between the extant literature and the answers to the above questions. Studies in the field of green credit policy focus mainly on its direct effects on polluting and non-polluting firms. For example, studies show that the policy can improve (weaken) eco-friendly (eco-unfriendly) firms’ financing conditions by adjusting the loan amount granted, loan interest, and loan maturity [5,13]. Second, the green credit policy can further impact corporate investment decisions, thereby resulting in more green investment activities [6,14]. Finally, various authors discuss the macro effects of the policy and find that pollution levels of the supply chain and the whole industry are lower after the implementation of the policy, and that industrial structure is optimized [15,16,17]. Few studies focus on environment-friendly manufacturing enterprises or the redistribution effect; thus, further insight is urgently needed.

In addition, the foundations of the redistribution effect remain to be elucidated. China’s green credit policy is comprehensive: although the policymakers are several central government departments, its regulatory objects include commercial banks, local governments, and enterprises [18]. Their heterogeneity can affect the redistribution effect. For SMEMEs, the most important element in their loan financing process is information [19]; hence, the level of information asymmetry between company and investor is a key factor that influences the redistribution mechanism. Moreover, regional financial development determines the capability and efficiency of local banks [20], which can further impact their decision-making and help SMEMEs obtain more loans in the context of the green credit policy. In addition, environmental investment, which is the primary method used by local governments for market intervention, can also provide additional revenue for banks. However, such support may only be offered to LEMEs, which maintain better relationships with local governments. Thus, the local governments’ investments may stimulate or mitigate the redistribution effect of the policy [21]. Based on these analyses, a new question arises: are corporate information asymmetry, regional financial development, and local government environmental investment the foundations of the green credit policy?

Therefore, in response to the research gap and ecological modernization theory, we explore the above questions using data on 155 Chinese environment-friendly manufacturing enterprises and 313 non-ecological enterprises listed on the Shanghai and Shenzhen stock exchanges from 2007 to 2019. We classify SMEMEs and LEMEs according to China’s classification standards of small and medium-sized enterprises (SMEs) and use a difference in difference (DID) model and a difference in difference in difference model (DDD) to evaluate our preliminary opposing hypotheses. Since the implementation of the green credit policy is affected by corporate information conditions, regional financial development, and local government environmental investment, we also discuss the heterogeneity of these three potential foundations.

The theoretical and practical contributions of this study are threefold: first, considering the relationship between the green credit policy and the characteristics of environment-friendly manufacturing enterprises with various scale levels, we expand the research into China’s green credit policy and the field of green finance, e.g., Xing et al. (2020) and Xu and Li (2020) [4,5]. To the best of our knowledge, our study is the first to consider an additional influence (redistribution effect) in the green financial policy literature. Second, this paper, which is based on ecological modernization theory, shows that green credit policy can consider both economic development and environmental protection. The findings support the embodiment of the core viewpoint of ecological modernization in green credit policy, magnify the theoretical significance of Pataki (2009) [22], and provide small and medium-sized environment-friendly manufacturing enterprises with more theoretical support for obtaining bank loans and financial resources. Finally, in combination with corporate information asymmetry, regional financial development, and local government environmental investment, we explore the foundations and moderators of the green credit policy’s redistribution effect, supplement the literature in these fields, and establish fundamental results regarding the optimization of the green credit policy worldwide.

The remainder of this study is organized as follows: Section 2 details the background and hypotheses. Section 3 presents the methodology. Section 4 presents the preliminary empirical results. Section 5 analyses the heterogeneities and foundations of the redistribution mechanism, and the final section presents the conclusions and implications of this study.

## 2. Background and Hypotheses

### 2.1. Ecological Modernization Theory and the Green Credit Policy

According to ecological modernization theory, economic development can flourish while realizing environmental protection. These two aspects never conflict if modern ideas, technologies, or measures are utilized [22]. In the field of environmental regulation, the concept of ecological modernization is also implemented widely in China’s environmental protection policies: in particular, environmental policies should not only help the environment recover but also establish a supervision system that does not impede economic development [23]. China’s green credit policy was designed in 2007 and formally implemented in 2012 by several central government departments, which included the Ministry of Environmental Protection and the China Banking Regulatory Commission. It is a comprehensive policy that focuses on the loan market and is a typical embodiment of ecological modernization. Regarding environmental protection, the green credit policy can increase the efficiency of natural resource utilization and reduce the level of industrial pollution by supporting environment-friendly manufacturing enterprises while restraining the financing of highly polluting companies [6]. Regarding economic development, the supported environment-friendly manufacturing enterprises can grow rapidly due to the availability of abundant financial resources. Thus, traditional enterprises will change their business models into ecological friendly models, and environment-friendly manufacturing start-ups will be established; consequently, ecological products and services will be enriched, and a new area of economic growth will be discovered [15,24,25].

Various factors can affect the efficiency of China’s green credit policy. The key requirement for policy success is the distribution of loan resources [4]. However, in the stage of policy design, namely, from 2007 to 2011, authors discussed the potential disadvantages of the policy implementation. First, the information asymmetry between banks and enterprises is too difficult to resolve, especially for environment-friendly manufacturing enterprises, since the information on environmental protection—the principal business of these firms—is usually not disclosed in financial statements [18]. Theoretically, banks can only obtain this type of information via on-the-spot investigation or data support from the government [26], but use of these complex mechanisms will substantially reduce banks’ enthusiasm for information searching and loan granting, resulting in credit rationing and policy efficiency loss, as banks have the problem of path dependence: They are more likely to invest in enterprises that maintain satisfactory relationships with them than in firms with better environmental performance. In addition, for economic stability, the local government may conspire with local enterprises and provide false environmental protection information to evade the investigation of banks, referred to as greenwashing, to help firms obtain more loans, thereby leading to the failure of the green credit policy [27,28].

Nevertheless, recent empirical studies show that the green credit policy indeed achieves environmental protection and the distribution of loan resources; hence, the policy is successful in general. For instance, in line with the view that banks will reduce financing support for highly polluting enterprises to avoid legitimate environmental risks [29], Liu et al. (2019) show that the loan amount and maturity for China’s highly polluting enterprises are lower after the implementation of the green credit policy [13]. Based on these results, Xing et al. (2020) demonstrate that the financing constraints of enterprises with higher green performance are alleviated by the green credit policy [4]. Meanwhile, He et al. (2019) suggest that regional industrial pollution decreased after the implementation of the policy, which promoted the development of a green economy [6]. Thus, the green credit policy aligns with the core perspective of ecological modernization theory. Financing resources are extended preferentially to environment-friendly manufacturing enterprises, thereby realizing the objectives of environmental protection and development of the green economy by relaxing their financing constraints.

However, the disadvantages of the green credit policy may remain for small and medium-sized environment-friendly manufacturing enterprises, even though they are playing key roles in green economic development, environmental protection, and ecological innovation [6,8,21]. Traditionally, SMEs are more flexible and efficient [8], but they face tighter financing constraints due to information asymmetry and lack of collateral [30]. Therefore, the effects of China’s green credit policy on loan distribution among firms that differ in terms of scale, i.e., the redistribution effect, remain to be determined. In this study, we attempt to analyze the two potential opposing paths of the redistribution mechanism and conduct an empirical study to test our hypotheses.

### 2.2. The Redistribution of the Green Credit Policy: Opposing Paths

#### 2.2.1. Positive Perspective

As discussed above, two potential opposing paths can explain the redistribution effect of China’s green credit policy. According to the positive perspective, SMEMEs can obtain more loans after the implementation of the green credit policy since these firms have more advantages in terms of flexibility and efficiency but a lower possibility of greenwashing. Furthermore, local governments and financial institutions are willing to directly help SMEMEs under the guidelines of the policy.

First, SMEMEs can realize higher environmental and financial performance due to their higher operational efficiency. Previous studies show that large firms have more resources due to monopoly, political correlation, and economies of scale [31,32]. However, there are substantial problems in large enterprises, e.g., lower operational efficiency, complicated decision-making processes, and high resource redundancy [8,33]. Moreover, large environment-friendly manufacturing enterprises are typically transformed from other traditional industries; hence, they may have stranded assets, which will cause problems of path dependency and impede their R&D activities and production of environmental protection products [34]. In the context of the green credit policy, small and medium-sized environment-friendly manufacturing enterprises are more effective even under limited resource endowment. SMEMEs can realize higher environmental performance, for example, with lower emissions, more targeted green investment, and superior green products and process innovation [35,36]. Thus, banks grant loans to firms that can satisfy the environmental protection requirement of the policy. The financial performance of SMEMEs can also be improved. For example, with higher production efficiency, the ecological products and services of SMEMEs better satisfy the requirements of the market and consumers, which increases their revenue and corporate value and attracts investors with larger and stable earnings [37]. In summary, after the implementation of the green credit policy, banks can consider both environmental and economic performance by granting loans to SMEMEs, not only to realize the environmental protection objective but also to reduce default risk caused by insufficient future cash flow, in line with ecological modernization theory. Accordingly, loan resources should be redistributed from large environment-friendly manufacturing enterprises to small and medium-sized enterprises.

Second, for banks, SMEMEs’ environmental information may be more reliable than that of LEMEs since LEMEs are more likely to conduct greenwashing by conspiring with local governments. In China, large firms are typically important sources of revenue and taxes for local governments [38]. Local governments are more willing to help LEMEs acquire loans. As discussed above, after the implementation of the green credit policy, banks have used various methods to obtain corporate environmental protection information, among which consulting with local governments has been essential [26]. However, banks worry about the risks posed by such political connections. They are more cautious when analyzing LEME information from local governments because it may have been embellished or altered, namely, greenwashed [39]. This risk can be classified as legitimate. If greenwashing behavior is detected in the future, the relevant banks, as the main investors, will be punished by the central government or banking supervision departments. For instance, in 2017, the Banking Regulatory Bureau of Jiangxi Province reported cases that involved large firms obtaining bank loans by greenwashing. It warned several banks and called on them to work seriously to identify greenwashing. More importantly, as a small number of regional governments have had such problems, it is difficult for banks to trust information in other regions even if it has not been manipulated, which is the phenomenon of adverse selection [40]. Therefore, to avoid the impact of legitimate risks on their operation, banks will reduce support for LEMEs. They will extend more financing resources to SMEMEs for which the information provided to increase their loan amount has a lower likelihood of being greenwashed.

Finally, China’s central government and financial institutions have established several policies in combination with supporting SMEs and the green credit policy. In early 2019, the Ministry of Ecology and the Environment and the All-China Federation of Industry and Commerce jointly issued the “Opinions on supporting and serving the green development of private enterprises”, where the green finance policies for small and medium-sized and private environment-friendly manufacturing enterprises are important. In practice, many banks in China have explored the interaction between the financing of small and medium-sized enterprises and the green credit policy. For example, the Industrial and Commercial Bank of China, the largest commercial bank in China, has focused on SMEMEs since 2012, establishing a channel for obtaining corporate green information from government departments of environmental protection, and strengthened marketing support for SMEMEs. These cases demonstrate that green credit has a direct policy impact on the financing of SMEMEs.

In summary, according to the above discussion, China’s green credit policy has a redistribution effect on environment-friendly manufacturing firms of various sizes, and SMEMEs can benefit more than LEMEs, namely, banks indeed value SMEMEs. As the policy focuses primarily on the loan amounts that are granted to the firms [4], we establish the first positive hypothesis:

**Hypothesis** **1 (H1).** *Compared with large environment-friendly manufacturing enterprises, the green credit policy provides more loans for small and medium-sized environment-friendly manufacturing enterprises, i.e., it has a redistributive effect among firms of different scale.*

#### 2.2.2. Negative Perspective

Several difficulties are encountered with the above redistribution effect: In practice, the double spillover effects of green innovation, information asymmetry, and failure of government support increase the uncertainty of the policy.

First, although SMEMEs have higher operational efficiency, it is difficult to balance the environmental spillover effect and the technology spillover effect due to the lack of resources. The market for China’s eco-friendly products was established relatively late; hence, consumer demands change frequently. Environment-friendly manufacturing enterprises must engage in green innovation to maintain their competitive advantages [41]. In contrast to traditional innovation, green innovation should realize the environmental spillover effect to improve environmental conditions. Nevertheless, pioneering innovative enterprises will inevitably be imitated by other firms, thereby causing the problem of technological spillover [42]. Therefore, governments provide many methods, e.g., government subsidies, intellectual property laws, and patent registration support, for environment-friendly manufacturing enterprises to avoid the market failure caused by this double spillover effect [43]. However, SMEMEs have difficulty obtaining these types of support due to their small size, which reduces investors’ revenue expectations [44]. In this case, the green credit policy cannot guide the financial institutions to grant loans to SMEMEs and cannot increase their loan amounts.

Second, even if the level of greenwashing of SMEMEs is low, substantial credit information asymmetry remains between firms and banks. The green credit policy does not establish standards for assessing corporate credit conditions [4]. Therefore, banks that play a leading role in this policy often call for collateral to control the credit risks [18]. However, the high-value assets of environment-friendly manufacturing enterprises, especially of SMEMEs, are typically intangible assets related to environmental protection technology, which are not preferred by banks because of their opacity [18]. Nonetheless, LEMEs have abundant tangible assets due to their larger scale and resource redundancy; hence, they can be granted higher credit levels by offering more tangible collateral. In contrast, SMEMEs, due to failure to maintain satisfactory relations with banks and lack of collateral, often fail to obtain more loans [44]. It is concluded that the environmental information advantages of SMEMEs may be mitigated by the credit information disadvantages and, thus hinder their acquisition of loans.

Finally, although the central government and various banks desire to directly support SMEMEs via the policy, problems remain with the unbalanced allocation of loans. Communication and cooperation between SMEMEs and local governments are much less extensive, which reduces the level of industrial clusters; hence, green credit policy information cannot be fully spread to every small and medium-sized firm. For example, the Chengdu Federation of Industry and Commerce stated in 2019 that the level of the green industry cluster was too low and that some small environment-friendly manufacturing enterprises had no access to green credit due to communication deficiencies. Moreover, the standards for the implementation of the policy differ among banks. As a result, the implementation of the green credit policy is independent and inefficient [26]. SMEMEs still face credit rationing, which impedes their loan acquisition.

According to the discussion above, we establish the opposing hypothesis:

**Hypothesis** **2 (H2).** *Compared with large environment-friendly manufacturing enterprises, the green credit policy does not provide more loans for small and medium-sized environment-friendly manufacturing enterprises*.

## 3. Methodology

### 3.1. Data and Models

The sample for our research is 468 enterprises listed in Shanghai and Shenzhen stock exchanges, including 155 environment-friendly manufacturing enterprises and 313 non-ecological enterprises. According to Al-Tuwaijri et al. (2004), Shen et al., (2020), and Wang et al. (2020), environment-friendly manufacturing enterprises refer to firms whose main business are ecological products production [7,12,45]. Based on the corporate annual report and the industry classification of the listed companies, which is developed by Tonghuashun Finance and Economic, one of the most influential financial analysis companies in China, we manually analyze every firm‘s main business to determine whether it is an environment-friendly manufacturing enterprise. Financial data for the sample were collected from the China Stock Market and Accounting Research Database (CSMAR). Regional data were gathered from the National Bureau of Statistics of China (NBSC) and China’s Provincial Marketization Index Report.

Our study focuses on environment-friendly manufacturing enterprises of various sizes. Therefore, we further divide the total sample into SMEs and large firms. We utilize listed SMEs for three reasons: first, since we use panel data to explore the impact of the policy, the sample of listed firms is more available and accurate. Second, the financing frameworks of listed SMEs are in line with the conventional framework, as developed by Berger and Udell (2006) [46]. For example, similar to unlisted firms, China’s listed SMEs rely on mortgages (fixed-asset lending or asset-based lending), but the large listed firms can obtain more credit loans (financial statement lending) [47]. Thus, the use of the listed SMEs does not conflict with our statements and is effective for evaluating the above hypotheses. Finally, the listed SMEs and large firms differ substantially in terms of general features. China’s listed SMEs are mainly listed in the SME board, a key component of the Shenzhen Stock Exchange, which was launched in 2004. This board accepts the listing application of small and medium-scale firms only, for which the listing standards are less stringent than those of the main board, e.g., a lower profitability requirement, lower size requirement, and more relaxed corporate governance requirement. Therefore, similar to conventional SMEs, the listed SMEs are significantly smaller than the large firms, and the proportion of stated-owned enterprises among them is much lower, but their operational efficiency is higher [48]. Based on these differences, it is concluded that the sample of listed SMEs is appropriate.

As we discussed, the classification of SMEs is based on the sample firms listed in China’s SME board. However, some companies that are listed on this board can develop fast and grow into large firms. Thus, referring to Hou and Li (2019) and Yang et al. (2019), we further exclude the large firm sample from the SME board according to the Regulations on Classification Standards of Small and Medium-sized Enterprises [47,48]. For our research, we examine the period 2007 to 2019 since the green credit policy was designed in 2007 but formally implemented in 2012 and the loan conditions of environment-friendly manufacturing enterprises may have been different prior to 2007. Finally, 3895 firm-year observations (159 SMEMEs, 1137 LEMEs, 609 conventional SMEs, and 1990 conventional large firms) are obtained.

As the green credit policy can be regarded as an exogenous shock, the quasi-natural experiment method is more efficient to evaluate the policy impact [13]. Previous empirical studies on the policy mainly used the difference in difference (DID) model, which includes treated group (impacted by the policy) and controlled group (unaffected by the policy) [13,17,27]. Theoretically, the DID model is a typical quasi-natural experiment tool that can detect the effect of a policy by comparing different groups in different periods [27]. Thus, we also select the DID model as the first method to test our hypotheses. The DID model for our research is built as equation (1), where Amount and AmountFlow are the dependent variables, Treat is a dummy variable implying the treated group firm (SMEMEs), Post represents the affected period (2012 and after), and Controls represents the collection of control variables. The sample for the DID model included the environment-friendly manufacturing enterprises only. According to the model specification, Treat is the difference between groups (the first difference), Post is the difference between periods (the second difference). Therefore, the double interaction (Treat × Post) is the difference in difference (the difference between the first and second differences), and the key to our hypotheses, i.e., the real impact of the policy. The magnitude of a significantly positive (negative) coefficient of the double interaction indicates SMEMEs obtain corresponding more (less) loans than LEMEs, and the hypothesis H1 (H2) is supported.

However, although we presume SMEMEs are the most affected firms, all environment-friendly manufacturing enterprises can be affected by the green credit policy, hence the redistribution effect illustrated by the DID model may be attributed to other policies. According to Cai et al. (2016) [49], this problem can be mitigated by adding another group, that is, the difference in difference in difference (DDD) model. We establish the following DDD model to solve this bias and improve the robustness of our findings (Equation (2)). In this DDD model, we add a new dummy variable, i.e., Group, which is equal to 1 if the firm is an environment-friendly manufacturing enterprise. Accordingly, the observations of non-ecological manufacturing enterprises, which cannot be affected by the green credit policy, are supplemented to the sample. The key to the hypotheses become the triple interaction of the DDD model (Treat × Post × Group). Its coefficient suggests the loan excess or shortage of SMEMEs after excluding the difference between environment-friendly manufacturing enterprise and non-ecological enterprise, and should be significantly positive if SMEMEs obtain more loans due to the green credit policy (the redistribution effect, which corresponds to H1), but insignificant if H2 is supported.

At the same time, our models and samples may be faced with the problems of heteroscedasticity and autocorrelation. Following previous literature [50], we use the robust standard error of firm-level clustering in all regressions to avoid these problems.

(1) $$\begin{array}{cc} Amount\;/\;AmountFlow_{i,t} & =\;\alpha \;+\;\beta_{1}\times \;Treat_{i,t}\times \;Post_{i,t}+\;\beta_{2}\times \;Treat_{i,t}+\;\beta_{3}\times \;Post_{i,t}\\ & \;+\;Controls_{i,t}+\;\varepsilon_{i,t} \end{array}$$

(2) $$\begin{array}{cc} Amount\;/\;AmountFlow_{i,t} & =\alpha \;+\;\beta_{1}\times \;Treat_{i,t}\times \;Post_{i,t}\times \;Group_{i,t}+\;\beta_{2}\times \;Treat_{i,t}\times \;Post_{i,t}\\ & \;+\;\beta_{3}\times \;Treat_{i,t}\times \;Group_{i,t}+\;\beta_{4}\times \;Post_{i,t}\times \;Group_{i,t}\\ & \;+\;\gamma_{1}\times \;Treat_{i,t}+\;\gamma_{2}\times \;Post_{i,t}+\;\gamma_{3}\times \;Group_{i,t}\\ & \;+\;Controls_{i,t}+\;\varepsilon_{i,t} \end{array}$$

### 3.2. Variables

Loan availability is the key issue of small financing [46]. Therefore, for SMEMEs, the green credit policy mainly impacts the loan amount rather than other loan contract terms such as interest rate or maturity. Thus, consistent with Liu et al. (2019) and Xing et al. (2020) [4,13], we utilize two indicators to measure corporate loan size, which are the dependent variables: first, Amount is the current total bank loans that the firm holds divided by the total debt. Second, AmountFlow is the amount of new bank loans that are granted in the corresponding year divided by the total debt.

As we discussed above, our independent variables include Treat, Post, and Group. Treat is a dummy variable that equals 1 if a firm is an SMEME (namely, if it belongs to the treated group) but equals 0 if it is an LEME (in the control group). Post is a dummy variable, which equals 1 if the time is within the range of 2012 to 2019 (impacted period) and 0 otherwise (preimpact period). Group is also a dummy variable that equals 1 if the firm is an environment-friendly manufacturing enterprise.

We also employ several control variables that can affect firms’ loan amounts based on previous studies regarding bank loans and enterprise financing [4,51,52].

First, we control various corporate financial and operational characteristics, which include (1) corporate size (Size), which is the natural logarithm of the total assets; (2) corporate age (Age), which is the natural logarithm of the number of years since the company was established; (3) ownership, or stated-owned enterprises (SOE), which equals 1 if the firm is a state-owned enterprise; (4) financial performance, or the return on sale (ROS), which is equal to the net profit divided by the operating revenue; (5) financial leverage (Leverage), which is the asset-liability ratio; (6) growth ability (Growth), which is the growth rate of the operating revenue in the year; (7) tangibility of assets, or the ratio of property, plant, and equipment (PPE), which equals the fixed assets divided by the total assets; (8) investment level (Invest), which is the ratio of the investment expenditure to the total assets; and (9) cash holding (Cash), which equals the cash amount divided by the total assets.

Second, we control for several corporate governance attributes: (1) agency cost (AgntCost), which is the sum of the selling expenses and administrative expenses divided by the total assets; (2) the chairman’s power (Power), which is a dummy variable that equals 1 if the chairman is also the chief executive; (3) the proportion of independent directors (Indpdt), which is the proportion of independent directors relative to all board members; (4) the executive shareholding (MagHold), which is the shareholding ratio of senior executives; (5) ownership concentration (Top), which is the shareholding ratio of the largest shareholder; and (6) equity control separation (Separate), which is the percentage of equity (ownership) and control right separation.

Finally, we consider the effects of other financing methods and establish two variables: The first is equity financing (Equity), which is the proportion of the equity financing amount relative to the total assets in the current year. The other is bond financing (Bond), which is the proportion of the bond financing amount relative to total debt in the current year.

In addition, we use a series of dummy variables to control the region fixed effects (Province), industry fixed effects (Industry), and time fixed effects (Year).

The specifications of all variables are presented in Appendix A and Table A1.

### 3.3. Summary Statistics

Summary statistics for our sample are presented in Table 1, where Panel A shows the total sample, Panel B shows the sample of environment-friendly manufacturing enterprises, and Panel C shows the loan characteristics between Enterprises of different size in different years. According to Panel A, the Size, Age, and SOE of SMEs are all smaller than large firms, implying large firms are five times bigger, three years older, and more likely to be state-owned than SMEs on average. The Amount and AmountFlow of SMEs of the total sample are also lower than for large firms, indicating SMEs find it harder to obtain bank loans. However, in Panel B, the Amount of SMEMEs exceeds that of LEMEs but the AmountFlow is smaller. This may be due to the green credit policy and thus supports our hypothesis H1. However, some variables confirm the financing constraints for SMEs. For example, the ROS, Leverage, and Growth of SMEMEs are smaller, but Cash is higher. Small firms must hold more cash or loans for emergencies since they cannot acquire loans as rapidly and immediately as large enterprises do, which is referred to as a precautionary savings motive [53]. Similarly, the Equity of SMEMEs is larger but Bond is smaller; these can be attributed to the higher financing barriers. Consequently, SMEMEs can only acquire financing using equity. In addition, we find that SMEMEs have more independent directors and higher executive shareholding but also pay higher agency costs. In Panel C, the mean proportion of Amount to debt of the total sample is approximately 25%, and the proportion of AmountFlow is about 30%, both of them fluctuate in recent years. Almost all mean values in different years for large firms are larger than those for SMEs, indicating that SMEs are faced with higher barriers in applying for loans. However, the summary statistics only provide an overview of the sample, with the accurate impact of the green credit policy to be revealed by further econometric analyses. Thus, in the next section, we use the established models and regression analysis to explore the redistributive effects of the policy.

## 4. Preliminary Empirical Results

### 4.1. Preliminary Regression Results

We first test the direct effects of the main independent variables (Treat, Post, and Group) to connect the summary statistics with the regression analysis of DID and DDD models. The regression results of the direct effects are shown in Table 2, where columns (1) to (4) are based on the total sample, and columns (5) to (8) are the results of the environment-friendly manufacturing enterprises sample. Odd columns are pooled regressions, and even columns are fixed effect regressions.

As the summary statistics show, the mean value differences of bank loans between the treated group (SMEs) and untreated group (large firms) are positive except Amount in Table 1, Panel B. In Table 2, we retest these phenomena. According to columns (1) to (4), we find that all coefficients of Treat are significantly negative, which support the results of the total sample summary statistics, implying SMEs hold fewer loans. The coefficients of Group are positive, meaning environment-friendly enterprises can obtain more loans. This result corresponds with Table 1 where the mean values of Amount and AmountFlow in Panel A are less than those in Panel B. In columns (5) to (8), the effects of Treat on AmountFlow are still negative (columns (7) and (8)), while the coefficients are insignificant when the dependent variable is Amount (columns (5) and (6)). They are different from the summary statistics where the Diff. of Amount in Panel B is negative. A possible explanation is that the summary statistics show only univariate mean value differences that may face some bias. In this case, the regression results are more reliable [47]. Nevertheless, the current regression results are in line with the summary statistics because they show SMEMEs may not obtain fewer loans than LEMEs. Specifically, the insignificant coefficients of Treat suggest the loan levels of SMEMEs are near to LEMEs, which are originally lower as columns (1) to (4) illustrate. As we conjectured, this can be attributed to the green credit policy. This viewpoint can be further explained in combination with the coefficients of the policy implementation period variable (Post), that is, even though the corporate accesses to bank loans become narrower in recent years (the coefficients of Post are significantly negative), SMEMEs can obtain relatively more loans compared to other companies. Thus, the green credit policy should be a main reason.

In summary, the direct effects of three main variables reconcile with the summary statistics to a considerable degree. However, such direct effects cannot efficiently measure the policy impact. Therefore, based on the direct effects, we further use the DID and DDD models to test our hypothesis more precisely.

The preliminary regression results for the DID and DDD models are shown in Table 3, where columns (1) and (2) are the results of the DID model, (3) and (4) are those of the DDD model. According to the results, all double interactions of DID models and all triple interactions of DDD models are significantly positive. Specifically, the coefficients of Post × Treat in the DID model equal 0.114 and 0.164 in columns (1) and (2), meaning that SMEMEs can obtain approximately 11.4% more total loans and 16.4% more loan flow (divide by debt) than LEMEs because of the green credit policy. Columns (3) and (4) indicate a similar phenomenon as the coefficients of Treat × Post × Group are 0.099 and 0.205, meaning that the percentages of total loans and loan flow of SMEMEs are 9.9% and 20.5% higher than LEMEs even if the differences between environment-friendly enterprises and non-ecological enterprises are excluded. Hence, the DID and DDD models illustrate a consistent result, that the total loan amount and loan flow of SMEMEs are improved after the implementation of China’s green credit policy, and the redistribution effect indeed occurs in environmental enterprises with various scales. Thus, hypothesis H1 is supported, and hypothesis H2 is rejected.

The control variables are also significant. For example, Size is negatively correlated with AmountFlow because large firms typically prefer cheaper financing methods (e.g., internal financing or business credit) over bank loans. The coefficients of Invest are significantly positive, which can be attributed to the demand for loans by these firms being higher than those of their counterparts, which engage in fewer investment activities. AgntCost is negatively related to the dependent variables; hence, banks are unwilling to grant loans to enterprises with severe agency problems. However, in the total sample, Cash, Age, Growth and Top are significantly negative but insignificant for the environment-friendly manufacturing firms. That may be because general firms with higher cash holding, age, growth capability and shareholding concentration face lower financing constraints, but such constraints cannot be solved in environment-friendly manufacturing enterprises. In addition, we find that the coefficients of Equity are positive but those of Bond are negative, which may be due to pecking order theory, namely, enterprises prefer debt financing to equity financing [54]. Therefore, if a firm has a higher level of equity financing, it should have much higher fund demands and have already obtained the maximum loan amount that it can acquire. However, a company may select one of the two debt financing measures (loan or bond) because they are homologous, and more bonds will crowd out the corporate loan amount.

### 4.2. Robustness Tests

The DID and DDD models above and the variety of dependent variables already show good robustness. However, the DID model is still faced with some problems. Therefore, we utilize three methods to improve the robustness of our findings from the DID model.

Firstly, the key assumption of the DID model is parallel trends, namely, that the trends of the treated and control groups are similar prior to the impact of the policy. Therefore, according to Li et al. (2016) [55], we use the following event study model (Equation (3)) to evaluate the parallel trends.

(3) $$\begin{array}{cc} Amount/AmountFlow_{i,t} & =\alpha +\sum_{j=2}^{4}\beta {}_{j}\times Before_{i,j}+\sum_{k=1}^{7}\gamma {}_{k}\times After{}_{i,k}+\delta \times Current_{i}\\ & \;+\theta \times Treat_{i,t}\times T_{i}+Controls_{i,t}+\varepsilon_{i,t} \end{array}$$

In Equation (3), Before is a series of dummy variables that equal 1 if the treated group firm observation is made in the jth year before the implementation of the policy and otherwise equal 0. Similarly, After is a dummy variable of the treated group firm observation in the kth year after the implementation of the policy. Current is a dummy variable of the treatment group firm observation in 2012, and T is a continuous variable that specifies the year. If our sample is consistent with the parallel trend assumption, all coefficients of β should be insignificant [55].

**Figure 1:**
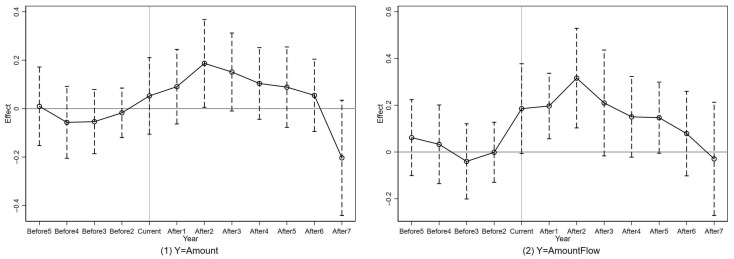
Parallel tests.

The results of the event study parallel tests are presented in Table 4 and Figure 1. We set 2011 (Before_1_) as the base year, which is omitted from the estimations to avoid collinearity [56]. According to the table, all coefficients of β (Before5 to Before2) are insignificant, but most coefficients of γ (After1 to After7) are significant. The figure presents the same results as all 95% confidence intervals of Before_j_ include the horizontal line of zero. These results demonstrate that the trends of the treated and control groups are parallel before the implementation of China’s green credit policy.

Second, we employ the propensity score matching difference in difference (PSM-DID) model. In practice, the number of SMEs is much larger than the number of large firms, but this proportion is smaller in our sample. Thus, the above findings suffer from sample bias. We use a matched sample to eliminate the bias. According to previous studies on SMEs [19,30], we select Size, Age, SOE, and Spread as the matching variables, where Spread is the bid-ask spread and an indicator of information asymmetry since SMEs are substantially smaller and younger than large firms and less owned by states but suffer from information asymmetry [57]. After 1-by-1 neighbor matching, we regress Equation (1) using the new sample. In addition, we evaluate the balance of our sample before and after matching.

The balance tests and regression results of PSM-DID are presented in Table 5. According to the tables, the sample is balanced after matching, and the interactions are significantly positive (0.131 and 0.247 respectively), that is, SMEMEs obtain 13.1% more total loans and 24.7% more loan flow than LEMEs. These coefficients are slightly larger than the original (11.4% and 16.4%); hence, our findings are supported and more significant when the sample bias problem is addressed.

**Figure 2:**
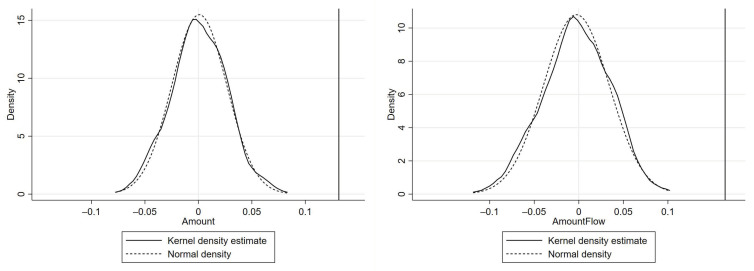
Placebo tests.

Finally, we conduct placebo tests of randomly assigned samples to evaluate the robustness of our results. According to Cai et al. (2016) [49], we distribute a corresponding proportion of SMEMEs and LEMEs randomly among our sample 200 times and record the coefficient of the DID interaction (Treat × Post) each time. The kernel density curves of the 200 coefficients are plotted in Figure 2. As the figure shows, both curves are in line with the normal density broadly and are away from the original coefficients (the vertical reference lines). Therefore, the placebo tests also support our findings and hypothesis H1.

## 5. The Heterogeneities and Foundations of the Redistribution Mechanism

### 5.1. Information Asymmetry, Financial Development, and Local Government Environmental Investment

The above findings show that the green credit policy has a redistribution effect and results in the provision of more loan resources to SMEMEs. However, this effect is not unchangeable. As we discussed previously, the redistribution effect will vary among contexts: in particular, hypothesis H2 may be supported in some cases. Theoretically, the policy mainly impacts three entities: enterprises, banks, and local governments [18]. First, for enterprises, a prominent problem of SMEMEs in the green credit policy is information asymmetry, which can directly influence their assessment by banks [19]. Second, banks, which are the implementers of the policy, determine whether environment-friendly manufacturing enterprises can obtain sufficient loans [18]. Finally, since China’s local governments are powerful supervisors of policy, their support also has a profound impact on green credit [4]. Based on these three perspectives, we further explore the heterogeneity of the redistribution effect under various corporate information asymmetry conditions, financial developments, and local government environmental investment levels.

For SMEMEs, even though the problem of asymmetric information is severe, there are paths for disclosing their environmental information. For example, firms can submit audited corporate social responsibility (CSR) reports every year, in which environmental protection is an important section. When they are applying for loans, these reports can provide essential certification [58]. Hence, the information asymmetry conditions differ among SMEMEs. Thus, in the context of the green credit policy, hypothesis H2 may be supported when the SMEMEs are faced with information asymmetry, but the companies with less severe communication problems can acquire more loans from banks since the information between banks and them is relatively balanced and the risks are lower, with the redistribution effect enhanced. Nonetheless, the information disadvantages of SMEMEs may be offset by the efficiency advantages. The above analyses and findings are evidence for this. As the preliminary results demonstrate, due to the redistribution effect, loans are preferentially extended to SMEMEs, which supports the positive view of high efficiency but does not support the negative view of information asymmetry. Based on these findings, it is difficult to conjecture whether SMEMEs with less asymmetric information can obtain more loans.

We argue that the redistribution effect of green credit policy is more significant in regions with mature financial development and does not occur in regions of low financial development (namely, H2 holds). The development of a more advanced financial system corresponds to a better marketization of banking. The relationship between banks and local governments will be symbiotic and interdependent [59]. Control by local government departments of financial institutions is weaker, and banks are more likely to grant loans to SMEMEs. The mature financial development results from abundant financial resources, such as more loan resources, more financial institutions, and higher efficiency; hence, the abilities of banks to serve SMEMEs is better, and the green credit policy cannot be a failure due to the richness of financial resources [20]. In addition, a sound financial system improves the development of financial technology (FinTech). Facilitated by big data, cloud computing and other technologies, the credit information of SMEMEs is more transparent [60]. In summary, in the regions with higher financial development, SMEMEs can obtain loans more easily due to the higher marketization level and abundant financial resources.

However, the role of local governments’ environmental investment is not clear. China’s administrative system determines that local government is not only the executor of economic regulation but also the institution responsible for environmental protection [61]. These responsibilities may have opposite impacts on the redistribution effect. Local governments must balance short-term and long-term objectives. From the perspective of short-term economic stability, governments are more inclined to intervene in the decision-making processes of banks. Therefore, they are more willing to support LEMEs, which have better relationships with local governments, and can also avoid increases in the unemployment rate and regional economic instability in the short term [62]. In this case, the green credit policy cannot help SMEMEs obtain more loans, and hypothesis H2 is supported. Nevertheless, if the local governments consider the requirements of ecological modernization and the sustainable development objective from a long-term perspective, they will build a fair business environment but not intervene in the decisions regarding green credit that are made by banks, and they will value the characteristics of high efficiency and satisfactory performance of SMEMEs. In this case, banks should provide SMEMEs with more policy convenience and, thus, more access to bank loans.

### 5.2. Further Empirical Analysis

We use three criteria to evaluate the three potential foundations that are specified above, and under each criterion, we divide our sample into two groups: For information asymmetry, the criterion is the corporate average daily ask-bid spread over the year, where high spread corresponds to severely asymmetric information [57]. We define the firms above the median of the sample in that year as the high information asymmetry group, and we assign the others to the low group. For financial development, we use the financial development and marketization index that was developed by Fan et al. (2019), which is an authoritative index for measuring China’s financial development level among provinces [59]. Firms that are above the median index in that year are classified into a high financial development group. For local governments’ environmental investment, we use the ratio of the regional environmental protection investment amount to the industrial added value as the criterion [4]. Similarly, enterprises are classified into the high local government investment group if they are located in a province in which the median investment level is exceeded. Based on these groups, we use the DID and DDD models that are expressed in Equations (1) and (2) to evaluate the potential foundations of the redistribution effect.

The results of the potential foundations are presented in Table 6, where Panels A, B, and C correspond to groups with high and low levels of information asymmetry, financial development, and local governments’ environmental investment, respectively. In every panel, columns (1) to (4) are the results of DID model, (5) to (8) of DDD model.

According to the table and Panel A, all the DID and the DDD models support the redistribution effect that occurs in both high and low information asymmetry conditions. Specifically, in the DID models, the effects of Treat × Post on Amount are 12.1% and 14.6% (columns (1) and (2)), and those on AmountFlow are 20.8% and 19.2% (column (3) and (4)). All the coefficients are near to the original. In the DDD models, the coefficients of the triple interactions are 0.113 and 0.155 on Amount (column (5) and (6)), and 0.315 and 0.218 on AmountFlow (column (7) and (8)). The effects of columns (6) and (7) are slightly larger than the original results of Table 3. Nevertheless, all the Chow tests indicate that there is no significant difference between high and low groups; the information disadvantages of SMEMEs are offset by efficiency advantages, and the corporate information may not be a foundation for the redistribution effect.

However, in Panel B, the key interactions of DID and DDD models are only significantly positive in the high financial development groups and significantly different from the low groups, which supports our conjecture that better regional financial conditions improve SMEMEs’ loan acquisition. All coefficients in high financial development groups are larger than the original and in the relatively lower group, the DID models indicate SMEMEs obtain 16.4% more total loans and 27.4% more loan flow than LEMEs because of the green credit policy (columns (1) and (3)). The DDD models show the effects become 14.3% and 29.5%, respectively, after eliminating the differences between environment-friendly manufacturing enterprises and non-ecological enterprises (columns (5) and (7)). This result also demonstrates that the redistribution effect is mitigated and hypothesis H2 is supported in the case of low financial development.

In addition, such interactions in Panel C are significantly positive in the low local government investment groups, but the Chow tests suggest that the difference is significant only for Amount. Indeed, in the low groups, the coefficients of the double interaction in column (4) and the triple interaction in column (8) are 0.153 and 0.214, which are close to the results of Table 3, but those in the high groups are larger than the original. This may be due to the long-term effects of local governments’ environmental investments: specifically, such investments typically impact firms for more than one year; hence, the long-term loan indicator (Amount) is more significant than the short-term indicator (AmountFlow). Nevertheless, similar to the mechanism of financial development, the redistribution effect can be negatively affected by local governments.

In summary, information asymmetry may not a foundation of the redistribution effect, but better financial development can enhance it. Local government investment mainly helps LEMEs; hence, it mitigates the redistribution effect.

## 6. Conclusions and Implications

### 6.1. Conclusions and Discussion

In this paper, we empirically investigate whether the green credit policy results in the provision of more loans to small and medium-sized environment-friendly manufacturing enterprises (SMEMEs) compared with large environment-friendly manufacturing enterprises (LEMEs), namely, the redistribution effect of the policy among firms of various scales. Based on ecological modernization theory and data on 155 listed environment-friendly manufacturing companies and 313 non- environment-friendly manufacturing companies of China, we obtain the following conclusions:

First, by using a difference in difference (DID) model and difference in difference in difference (DDD) model, we show that China’s green credit policy improves SMEMEs’ loan amounts by 9.9% to 11.4% more total loans and 16.4% to 20.5% more loan flow than LEMEs; hence, there is a redistribution effect of the green credit policy among enterprises of various sizes, and banks indeed value SMEMEs. This is because banks prefer the ecological modernization characteristics of SMEMEs, which include higher operational efficiency, less greenwashing, and more direct financial support, which can not only improve the ecology but also promote the economy. This finding contributes to the previous literature on green credit policy, in which the direct impacts of the policy on corporate bank loans, environmental investment, and environmental performance are the main considerations [6,13,16,17], while less consideration is given to the SMEMEs. Our conclusion provides a deeper insight into China’s green credit policy and, thus, can provide policymakers, banks, and enterprises with an additional reference for their decision making and the realization of the sustainability objective.

Second, we find that the conditions of information asymmetry between firms and banks are not a foundation of the redistribution effect, since the effects of the policy are similar in both high and low information asymmetry groups. This can be attributed to banks’ focus on SMEMEs’ higher efficiency and flexibility, as such advantages offset the disadvantage of asymmetric information. Therefore, regardless of the information conditions, SMEMEs can obtain loans of the same value as LEMEs. The finding provides important evidence that the green credit policy can help small firms with less communication access by overcoming information asymmetry problems and makes new progress regarding SME financing. Previous studies in this field mainly examine SMEs’ financing constraints that result from asymmetric information and propose direct solutions such as information disclosure and relational lending [19,30,54,59]. However, our empirical analysis provides an indirect path for SMEs to overcome the financing constraints and achieve the sustainable development.

Third, the results demonstrate that the redistribution effect only occurs in regions with higher financial development. SMEMEs can obtain approximately 5% additional total loans and 10% additional loan flow in well-developed regions. This heterogeneity is due to financial marketization and abundant finance resources: SMEMEs in well-developed regions suffer less from resource constraints and adverse selection and, thus, can acquire more loans due to the green credit policy. However, in underdeveloped regions, the lack of resources and policy failures cannot be solved by banks, and their disadvantages further decrease the probability of granting loans to SMEMEs. This finding shows that a better financial environment can promote the financing of small and medium-sized enterprises and establishes a link with green finance. Various authors have identified the importance of financial development from the aspects of the firm, region, and macroeconomy [20,63], but its impacts on corporate sustainable development and finance have been less discussed. To the best of our knowledge, only Yin et al. (2019) conjecture that financial development can enhance the performance of the environmental policy, but they do not empirically evaluate this viewpoint by utilizing a sample of enterprises [64]. Our conclusion fills this gap.

Finally, we find that the redistribution effect can also be mitigated by local governments’ environmental investment, especially in terms of the corporate total loan amount. SMEMEs in regions with lower investment levels obtain approximately 4% more total loans and 9% more loan flow than LEMEs. This is because SMEMEs have weaker connections with local governments. In contrast, LEMEs are typically state-owned and play key roles in local employment and economy stability; hence, although local governments provide more funds to green credit, the main recipients are LEMEs. This result also suggests that local government intervention in China’s green credit policy may reduce the marketization and efficiency of the policy and sustainable development. However, this conclusion provides an important reference for policymakers and contributes to the literature. Many studies examine the relationship between government regulation or intervention and corporate environmental behavior [16,17,23,41,62], but its impact on firms of various sizes in the context of the green credit policy has yet to be elucidated. Our study identifies this mechanism and provides insight into the relationship among the central government, local governments, and enterprises of China.

### 6.2. Practical Implications

The above conclusions have implications for environment-friendly manufacturing enterprises, financial institutions, and governments.

First, it is important to preserve the ecological modernization characteristics of SMEMEs, e.g., high efficiency and flexibility, in order to maintain the support of the green credit policy. SMEMEs should invest more financial resources in the main business of products, overcome the technical bottleneck in the current ecological industry, and strengthen market capacity. LEMEs should focus on increasing their operational efficiency, incorporating the business model and strategic arrangement of the higher-performing enterprises in the same industry, improving the quality of products and services, and reducing the dependence on local governments. For general start-ups, entering the environment-friendly manufacturing industry is reasonable. According to our findings, in the context of the green credit policy, small firms can obtain loans without high informational transparency; thus, their financing problem can be solved to a large extent.

Second, banks should pay more attention to the implementation of the green credit policy and its development. Since greenwashing is a significant factor that impedes access to loans by firms (especially LEMEs), banks must disclose the implementation of the green credit policy to the public on a timely basis to promote social supervision, reduce the occurrence of greenwashing, and develop a fair environment for firms of various sizes. In addition, banks should optimize their operation and increase their efficiency to increase the level of regional financial development and enhance the redistribution effect of the green credit policy. Moreover, they should reduce local government intervention moderately and realize the sustainability objectives of the policy and the high-quality development of ecological economies.

Finally, it is necessary for government departments to strengthen the optimization of the regional financial system and reduce intervention in banks’ decision-making processes. Our results demonstrate that the main driving factor of the redistribution effect of the green credit policy is financial development. As a powerful regulator of the local economy, local government departments should focus on strengthening regional financial construction and on increasing the recruitment and investment of financial institutions and infrastructure. In addition, local government departments should focus on long-term development and should increase financial support to all firms with prominent environmental performance rather than to companies with political connections. This should create an equal environment for various enterprises for the realization of both economic development and environmental protection.

### 6.3. Limitations

Several limitations of this study have been identified. For example, does the green credit policy have a redistribution effect on all environment-friendly manufacturing enterprises in all regions and industries? In addition, our sample is based on data for listed companies, which are large in scale even though we classify them according to a series of national standards. Thus, for unlisted firms, does the redistribution effect still occur? These questions are unanswered in this paper, but they provide potential directions for follow-up investigation.

## Figures and Tables

**Table 1:** Summary statistics.

**Panel A. Total Sample**									
**Variables**	**Summary Statistics**						**Difference between Groups**		
	**Obs**	**Mean**	**STD**		**Min**	**Max**	**Treat = 0**	**Treat = 1**	**Diff.**
Total Bank Loans (Amount)	3895	0.253	0.223		0.000	0.921	0.268	0.189	0.079 ***
New Granted Loans (AmountFlow)	3895	0.306	0.337		0.000	3.846	0.320	0.251	0.069 ***
Corporate Size (Size)	3895	21.650	1.226		10.842	27.146	21.989	20.270	1.718 ***
Corporate Age (Age)	3895	2.652	0.415		0.000	3.611	2.699	2.458	0.241 ***
State-owned Enterprises (SOE)	3895	0.326	0.469		0.000	1.000	0.380	0.108	0.272 ***
Return on Sale (ROS)	3895	0.083	0.234		−1.446	0.463	0.080	0.099	−0.019 **
Financial Leverage (Leverage)	3895	0.384	0.204		0.011	0.997	0.411	0.276	0.135 ***
Growth Ability (Growth)	3895	0.243	0.494		−0.573	2.978	0.250	0.217	0.033 *
Tangibility of Assets (PPE)	3895	0.255	0.170		0.000	0.893	0.261	0.230	0.031 ***
Investment Level (Invest)	3895	0.047	0.050		0.000	0.477	0.046	0.052	−0.006 ***
Cash Holding (Cash)	3895	0.258	0.183		0.000	1.000	0.229	0.375	−0.146 ***
Agency Cost (AgntCost)	3895	0.232	0.171		0.018	0.910	0.217	0.291	−0.074 ***
Chairman’s Power (Power)	3895	0.292	0.455		0.000	1.000	0.260	0.419	−0.159 ***
Independent Directors (Indpdt)	3895	0.375	0.056		0.000	0.667	0.374	0.379	−0.005 **
Shareholding of Executives (MagHold)	3895	0.153	0.207		0.000	0.790	0.124	0.273	−0.149 ***
Shareholding Concentration (Top)	3895	32.749	15.190		3.546	85.232	32.812	32.490	0.322
Equity-control Separation (Separate)	3895	3.511	6.103		0.000	38.847	3.618	3.076	0.542 **
New Acquired Equity Funds (Equity)	3895	0.061	0.143		−0.004	0.839	0.046	0.120	−0.074 ***
New Acquired Bond Funds (Bond)	3895	0.011	0.056		0.000	0.738	0.014	0.001	0.013 ***
**Panel B. Environment−Friendly Manufacturing Enterprises Sample**									
**Variables**	**Summary Statistics**						**Difference between Groups**		
	**Obs**	**Mean**	**STD**		**Min**	**Max**	**Treat = 0**	**Treat = 1**	**Diff.**
Amount	1296	0.352	0.202		0.006	0.831	0.347	0.383	−0.036 **
AmountFlow	1296	0.387	0.262		0.000	0.979	0.398	0.312	0.085 ***
Size	1296	22.034	1.174		18.276	26.191	22.226	20.665	1.561 ***
Age	1296	2.694	0.373		0.693	3.611	2.726	2.468	0.258 ***
SOE	1296	0.422	0.494		0.000	1.000	0.449	0.226	0.223 ***
ROS	1296	0.072	0.190		−1.446	0.462	0.078	0.033	0.045 ***
Leverage	1296	0.485	0.182		0.045	0.997	0.499	0.383	0.115 ***
Growth	1296	0.236	0.481		−0.573	2.978	0.249	0.138	0.111 ***
PPE	1296	0.348	0.158		0.003	0.893	0.351	0.326	0.025 *
Invest	1296	0.056	0.051		0.000	0.477	0.056	0.056	0.000
Cash	1296	0.178	0.124		0.001	0.801	0.169	0.247	−0.078 ***
AgntCost	1296	0.143	0.103		0.018	0.910	0.132	0.220	−0.087 ***
Power	1296	0.221	0.415		0.000	1.000	0.207	0.321	−0.114 ***
Indpdt	1296	0.367	0.059		0.000	0.600	0.366	0.376	−0.010 **
MagHold	1296	0.054	0.127		0.000	0.703	0.045	0.118	−0.072 ***
Top	1296	36.169	15.195		10.420	85.232	36.104	36.629	−0.525
Separate	1296	4.990	7.570		0.000	38.847	5.252	3.113	2.139 ***
Equity	1296	0.046	0.121		0.000	0.771	0.036	0.120	−0.084 ***
Bond	1296	0.018	0.069		0.000	0.738	0.020	0.002	0.018 ***
**Panel C. Loan Characteristics between Small and Medium-Sized Enterprise (SME) and Large Enterprise in Different Years**									
**Year**	**Obs**	**Amount**				**AmountFlow**			
		**Mean** **Overall**	**Mean** **Treat = 0**	**Mean** **Treat = 1**	**Diff.**	**Mean** **Overall**	**Mean** **Treat = 0**	**Mean** **Treat = 1**	**Diff.**
2007	129	0.366	0.384	0.313	0.071	0.385	0.409	0.317	0.091
2008	139	0.355	0.386	0.282	0.105 **	0.420	0.430	0.395	0.034
2009	165	0.321	0.339	0.286	0.053	0.451	0.440	0.473	−0.033
2010	227	0.269	0.305	0.211	0.094 ***	0.339	0.379	0.274	0.105 **
2011	272	0.242	0.279	0.179	0.100 ***	0.282	0.328	0.204	0.124 ***
2012	294	0.220	0.257	0.136	0.121 ***	0.290	0.321	0.222	0.100 ***
2013	310	0.240	0.266	0.167	0.099 ***	0.311	0.337	0.239	0.098 **
2014	327	0.242	0.254	0.186	0.068 **	0.298	0.311	0.239	0.072
2015	358	0.250	0.261	0.186	0.075 **	0.314	0.324	0.249	0.075
2016	414	0.222	0.229	0.169	0.060 *	0.277	0.286	0.206	0.080 *
2017	452	0.240	0.252	0.142	0.110 ***	0.282	0.297	0.158	0.140 ***
2018	461	0.256	0.266	0.153	0.113 ***	0.279	0.291	0.155	0.136 ***
2019	347	0.235	0.246	0.118	0.128 ***	0.274	0.284	0.170	0.115 *

Note: * *p* < 0.1, ** *p* < 0.05, and *** *p* < 0.01.

**Table 2:** Regression results of direct effects of main variables.

	Total Sample				Environment-Friendly Manufacturing Enterprises Sample			
	(1)	(2)	(3)	(4)	(5)	(6)	(7)	(8)
	Amount	Amount	Amount Flow	Amount Flow	Amount	Amount	Amount Flow	Amount Flow
Group	0.136 ***	0.125 ***	0.107 ***	0.124 ***				
	(8.04)	(5.14)	(4.70)	(3.70)				
Treat	−0.070 ***	−0.067 ***	−0.068 ***	−0.070 ***	0.016	−0.000	−0.102 ***	−0.080 **
	(−4.52)	(−4.59)	(−3.05)	(−3.32)	(0.47)	(−0.01)	(−2.76)	(−2.25)
Post	−0.057 ***	−0.090 ***	−0.073 ***	−0.089 **	−0.063 ***	−0.150 ***	−0.050 **	−0.102 **
	(−4.48)	(−4.06)	(−3.97)	(−2.58)	(−3.32)	(−4.31)	(−2.32)	(−2.39)
Province	No	Yes	No	Yes	No	Yes	No	Yes
Industry	No	Yes	No	Yes	No	Yes	No	Yes
Year	No	Yes	No	Yes	No	Yes	No	Yes
Constant	0.265 ***	0.362 ***	0.340 ***	0.413 ***	0.395 ***	0.453 ***	0.435 ***	0.479 ***
	(16.10)	(13.75)	(14.60)	(9.36)	(17.67)	(14.53)	(16.95)	(11.51)
*N*	3895	3895	3895	3895	1296	1296	1296	1296
Adj. R2	0.119	0.192	0.040	0.095	0.021	0.228	0.017	0.203

Note: *t*-statistics are presented in parentheses (corrected for heteroskedasticity and firm-level clustering), and *** *p* < 0.01, ** *p* < 0.05, and * *p* < 0.1 (two-tailed).

**Table 3:** Preliminary regression results for the difference in difference (DID) and difference in difference in difference (DDD) models.

	DID Model		DDD Model	
	(1)	(2)	(3)	(4)
	Amount	AmountFlow	Amount	AmountFlow
Treat × Post × Group			0.099 *	0.205 ***
			(1.83)	(2.87)
Treat × Post	0.114 **	0.164 ***	−0.002	−0.058
	(2.41)	(3.31)	(−0.08)	(−1.17)
Treat × Group			0.048	−0.109 *
			(1.10)	(−1.75)
Post × Group			−0.018	0.019
			(−0.67)	(0.48)
Treat	−0.021	−0.147 ***	−0.039	−0.008
	(−0.55)	(−3.50)	(−1.39)	(−0.18)
Post	−0.136 ***	−0.057	−0.017	0.033
	(−2.92)	(−0.95)	(−0.58)	(0.62)
Group			0.137 ***	0.158 **
			(2.89)	(2.44)
Size	−0.004	−0.027 *	−0.004	−0.018 *
	(−0.36)	(−1.93)	(−0.57)	(−1.74)
Age	−0.013	−0.008	−0.039 **	−0.083 ***
	(−0.34)	(−0.17)	(−2.28)	(−3.01)
SOE	−0.048	−0.054	−0.026	−0.019
	(−1.62)	(−1.50)	(−1.52)	(−0.60)
ROS	−0.020	−0.060	−0.009	−0.007
	(−0.48)	(−1.11)	(−0.45)	(−0.24)
Leverage	0.228 ***	0.125	0.278 ***	0.192 ***
	(3.68)	(1.53)	(7.36)	(3.80)
Growth	−0.004	0.002	−0.017 ***	−0.034 ***
	(−0.37)	(0.12)	(−2.79)	(−3.85)
PPE	0.022	0.097	−0.018	0.045
	(0.32)	(1.17)	(−0.42)	(0.68)
Invest	0.558 ***	0.625 ***	0.416 ***	0.669 ***
	(3.92)	(3.28)	(4.38)	(3.89)
Cash	−0.077	−0.104	−0.313 ***	−0.378 ***
	(−0.84)	(−0.95)	(−7.79)	(−6.64)
AgntCost	−0.242 ***	−0.413 ***	−0.141 ***	−0.169
	(−2.63)	(−3.66)	(−3.93)	(−1.31)
Power	0.006	−0.019	0.015	−0.005
	(0.29)	(−0.74)	(1.20)	(−0.30)
Indpdt	0.136	0.045	0.076	−0.012
	(1.01)	(0.27)	(0.83)	(−0.08)
MagHold	−0.019	0.110	−0.011	−0.049
	(−0.23)	(1.14)	(−0.30)	(−0.93)
Top	−0.001	−0.000	−0.002 ***	−0.002 **
	(−1.10)	(−0.46)	(−3.56)	(−2.23)
Separate	−0.002	−0.001	−0.001	−0.002
	(−1.39)	(−0.94)	(−0.95)	(−1.14)
Equity	0.144 ***	0.206 ***	0.100 ***	0.270 ***
	(2.66)	(2.68)	(4.23)	(4.85)
Bond	−0.280 ***	−0.308 ***	−0.186 ***	−0.100
	(−4.63)	(−4.11)	(−3.73)	(−1.35)
Province	Yes	Yes	Yes	Yes
Industry	Yes	Yes	Yes	Yes
Year	Yes	Yes	Yes	Yes
Constant	0.443 **	1.028 ***	0.558 ***	1.223 ***
	(1.98)	(3.53)	(3.24)	(4.67)
*N*	1296	1296	3895	3895
Adj. R2	0.339	0.329	0.376	0.202

Note: *t*-statistics are presented in parentheses (corrected for heteroskedasticity and firm-level clustering), and *** *p* < 0.01, ** *p* < 0.05, and * *p* < 0.1 (two-tailed).

**Table 4:** Regression results of parallel tests.

	(1)	(2)
	Amount	AmountFlow
Before5	0.010	0.062
	(0.12)	(0.75)
Before4	−0.057	0.033
	(−0.76)	(0.39)
Before3	−0.054	−0.040
	(−0.80)	(−0.49)
Before2	−0.017	−0.001
	(−0.34)	(−0.02)
Current	0.052	0.185 *
	(0.66)	(1.91)
After1	0.091	0.197 ***
	(1.16)	(2.77)
After2	0.187 **	0.316 ***
	(2.04)	(2.93)
After3	0.151 *	0.210 *
	(1.85)	(1.83)
After4	0.104	0.151 *
	(1.39)	(1.72)
After5	0.089	0.147 *
	(1.05)	(1.91)
After6	0.055	0.079
	(0.72)	(0.86)
After7	−0.203 *	−0.029
	(−1.69)	(−0.24)
Treat × T	0.000	−0.000 ***
	(0.05)	(−3.00)
Control Variables	Yes	Yes
Province	Yes	Yes
Industry	Yes	Yes
Year	Yes	Yes
Constant	0.540 **	1.062 ***
	(2.48)	(3.74)
*N*	1296	1296
Adj. R2	0.335	0.328

Note: *t*-statistics are presented in parentheses, and *** *p* < 0.01, ** *p* < 0.05, and * *p* < 0.1 (two-tailed).

**Table 5:** Results of propensity score matching difference in difference (PSM-DID) model.

**Panel A. Balanced Tests**					
**Variables**	**Match**	**Treated Group**	**Controlled Group**	**t**	***p*-Value**
Size	Unmatched	20.713	22.233	−16.72	0.000 ***
	Matched	20.713	20.667	0.55	0.584
Age	Unmatched	2.495	2.733	−7.40	0.000 ***
	Matched	2.495	2.507	−0.25	0.804
SOE	Unmatched	0.238	0.434	−4.56	0.000 ***
	Matched	0.238	0.197	0.85	0.398
Spread	Unmatched	0.048	0.041	5.89	0.000 ***
	Matched	0.048	0.048	−0.25	0.805
**Panel B. Regression Results**					
		**(1)**		**(2)**	
		**Amount**		**AmountFlow**	
Treat × Post		0.131 *		0.247 **	
		(1.90)		(2.39)	
Treat		−0.026		−0.288 ***	
		(−0.59)		(−4.83)	
Post		−0.346 **		−0.156	
		(−2.36)		(−0.82)	
Control Variables		Yes		Yes	
Province		Yes		Yes	
Industry		Yes		Yes	
Year		Yes		Yes	
Constant		−0.253		2.116 *	
		(−0.37)		(1.96)	
*N*		209		209	
Adj. R2		0.363		0.235	

Note: *t*-statistics are presented in parentheses (corrected for heteroskedasticity and firm-level clustering), and *** *p* < 0.01, ** *p* < 0.05, and * *p* < 0.1 (two-tailed).

**Table 6:** Regression results of further analysis.

**Panel A. Regression Results of Different Information Asymmetry Levels**								
	**DID Model**				**DDD Model**			
	**(1)**	**(2)**	**(3)**	**(4)**	**(5)**	**(6)**	**(7)**	**(8)**
	**High Amount**	**Low Amount**	**High Amount Flow**	**Low Amount Flow**	**High Amount**	**Low Amount**	**High Amount Flow**	**Low Amount Flow**
Treat × Post × Group					0.113 *	0.155 *	0.315 ***	0.218 **
					(1.91)	(1.85)	(3.01)	(2.07)
Treat × Post	0.121 **	0.146 *	0.208 ***	0.192 **	−0.007	−0.023	−0.097	−0.049
	(2.53)	(1.75)	(3.46)	(2.21)	(−0.18)	(−0.67)	(−1.14)	(−0.90)
Treat × Group					0.016	0.075	−0.248 ***	−0.074
					(0.30)	(1.23)	(−2.74)	(−0.80)
Post × Group					−0.032	−0.024	0.035	0.017
					(−0.86)	(−0.80)	(0.69)	(0.39)
Treat	−0.052	0.016	−0.200 ***	−0.111	−0.026	−0.027	0.047	−0.020
	(−1.34)	(0.28)	(−4.27)	(−1.52)	(−0.69)	(−0.84)	(0.61)	(−0.38)
Post	−0.108 *	−0.095 *	−0.064	0.061	0.038	−0.070 *	0.093	−0.008
	(−1.72)	(−1.66)	(−0.74)	(0.81)	(0.85)	(−1.70)	(1.18)	(−0.11)
Group					0.161 ***	0.111 *	0.170 **	0.144 *
					(3.02)	(1.84)	(2.13)	(1.85)
Control Variables	Yes	Yes	Yes	Yes	Yes	Yes	Yes	Yes
Province	Yes	Yes	Yes	Yes	Yes	Yes	Yes	Yes
Industry	Yes	Yes	Yes	Yes	Yes	Yes	Yes	Yes
Year	Yes	Yes	Yes	Yes	Yes	Yes	Yes	Yes
Constant	−0.146	0.641 **	0.519	1.355 ***	0.396 *	0.719 ***	1.384 ***	1.096 ***
	(−0.46)	(2.53)	(1.18)	(4.48)	(1.72)	(3.54)	(2.90)	(4.09)
*N*	609	658	609	658	1689	2050	1689	2050
Adj. R2	0.298	0.451	0.309	0.385	0.379	0.398	0.147	0.259
	**Diff.**		**Diff.**		**Diff.**		**Diff.**	
Difference	−0.025		0.016		−0.042		0.097	
*p*-value	0.695		0.899		0.759		0.446	
**Panel B. Regression Results of Different Financial Development Levels**								
	**DID Model**				**DDD Model**			
	**(1)**	**(2)**	**(3)**	**(4)**	**(5)**	**(6)**	**(7)**	**(8)**
	**High** **Amount**	**Low** **Amount**	**High** **Amount** **Flow**	**Low** **Amount** **Flow**	**High** **Amount**	**Low** **Amount**	**High** **Amount** **Flow**	**Low** **Amount** **Flow**
Treat × Post × Group					0.143 **	−0.062	0.295 ***	−0.133
					(2.19)	(−0.67)	(3.30)	(−1.16)
Treat × Post	0.164 ***	−0.066	0.274 ***	−0.145	0.001	−0.042	−0.059	−0.043
	(2.76)	(−0.92)	(4.17)	(−1.68)	(0.04)	(−1.04)	(−0.95)	(−0.65)
Treat × Group					0.046	0.004	−0.171 **	0.047
					(0.88)	(0.05)	(−2.25)	(0.43)
Post × Group					−0.031	0.002	−0.012	0.111
					(−1.00)	(0.04)	(−0.23)	(1.63)
Treat	−0.018	−0.046	−0.216 ***	0.006	−0.026	−0.046	0.007	−0.048
	(−0.40)	(−0.69)	(−4.26)	(0.06)	(−0.74)	(−1.13)	(0.12)	(−0.73)
Post	−0.099 **	−0.189	−0.010	−0.128	−0.005	−0.036	0.080	−0.103
	(−2.16)	(−1.49)	(−0.15)	(−0.73)	(−0.13)	(−0.59)	(1.12)	(−1.24)
Group					0.158 **	0.088	0.176 *	0.070
					(2.44)	(1.35)	(1.90)	(0.83)
Control Variables	Yes	Yes	Yes	Yes	Yes	Yes	Yes	Yes
Province	Yes	Yes	Yes	Yes	Yes	Yes	Yes	Yes
Industry	Yes	Yes	Yes	Yes	Yes	Yes	Yes	Yes
Year	Yes	Yes	Yes	Yes	Yes	Yes	Yes	Yes
Constant	−0.099 **	−0.189	−0.010	−0.128	0.506 **	0.864 ***	1.421 ***	0.838 **
	(−2.16)	(−1.49)	(−0.15)	(−0.73)	(2.54)	(2.82)	(4.69)	(2.17)
*N*	983	313	983	313	2651	1117	2651	1117
Adj. R2	0.377	0.456	0.349	0.403	0.385	0.411	0.197	0.266
	**Diff.**		**Diff.**		**Diff.**		**Diff.**	
Difference	0.230 ***		0.419 ***		0.205 ***		0.428 ***	
*p*-value	0.001		0.000		0.006		0.010	
**Panel C. Regression Results of Different Local Government Environmental Investment Levels**								
	**DID Model**				**DDD Model**			
	**(1)**	**(2)**	**(3)**	**(4)**	**(5)**	**(6)**	**(7)**	**(8)**
	**High** **Amount**	**Low** **Amount**	**High** **Amount** **Flow**	**Low** **Amount** **Flow**	**High** **Amount**	**Low** **Amount**	**High** **Amount** **Flow**	**Low** **Amount** **Flow**
Treat × Post × Group					−0.053	0.193 ***	0.219 *	0.214 ***
					(−0.56)	(2.98)	(1.82)	(2.61)
Treat × Post	−0.033	0.150 **	0.056	0.153 ***	0.015	−0.067	−0.067	−0.070
	(−0.33)	(2.59)	(0.49)	(2.70)	(0.42)	(−1.65)	(−1.00)	(−1.16)
Treat × Group					0.140 *	−0.025	−0.133	−0.074
					(1.90)	(−0.51)	(−1.45)	(−1.00)
Post × Group					0.013	−0.045	0.058	0.000
					(0.30)	(−1.26)	(0.94)	(0.01)
Treat	0.047	−0.052	−0.089	−0.155 ***	−0.039	−0.020	0.025	−0.077
	(0.72)	(−1.27)	(−1.40)	(−2.99)	(−1.03)	(−0.54)	(0.41)	(−1.34)
Post	−0.140 *	−0.107 **	−0.020	−0.035	−0.028	0.019	0.050	0.020
	(−1.73)	(−2.01)	(−0.21)	(−0.48)	(−0.66)	(0.43)	(0.59)	(0.33)
Group					−0.024	0.227 ***	−0.033	0.218 ***
					(−0.41)	(3.49)	(−0.38)	(2.62)
Control Variables	Yes	Yes	Yes	Yes	Yes	Yes	Yes	Yes
Province	Yes	Yes	Yes	Yes	Yes	Yes	Yes	Yes
Industry	Yes	Yes	Yes	Yes	Yes	Yes	Yes	Yes
Year	Yes	Yes	Yes	Yes	Yes	Yes	Yes	Yes
Constant	0.578 *	0.373	1.191 ***	0.843 ***	0.701 ***	0.718 ***	1.861 ***	1.012 ***
	(1.92)	(1.37)	(2.75)	(2.62)	(3.10)	(2.94)	(4.87)	(3.41)
*N*	377	919	377	919	1996	1772	1996	1772
Adj. R2	0.415	0.350	0.274	0.367	0.374	0.445	0.168	0.368
	**Diff.**		**Diff.**		**Diff.**		**Diff.**	
Difference	−0.183 **		−0.097		−0.246 ***		0.005	
*p*-value	0.009		0.319		0.000		0.447	

Note: *t*-statistics are presented in parentheses (corrected for heteroskedasticity and firm-level clustering), and *** *p* < 0.01, ** *p* < 0.05, and * *p* < 0.1 (two-tailed). The values of Diff. are the differences of double or triple interactions between the high and low groups (Chow tests).

## Data Availability

The data presented in this study are available on request from the corresponding author.

## Appendix A

**Table A1:** Specifications of variables.

Variable Type	Variable Name	Abbreviation	Specification
Dependent Variables	Total Bank Loans	Amount	The total bank loans that are held by the enterprise relative to the total debt.
	New Granted Loans	AmountFlow	The new bank loans that are granted in the corresponding year divided by the total debt.
Independent Variables	Treated Group	Treat	A dummy variable that equals 1 if the firm is an SME and 0 otherwise.
	Policy Implementation	Post	A dummy variable that equals 1 if the firm is observed on or after 2012 and 0 otherwise.
	Environment-friendly Manufacturing Enterprises	Group	A dummy variable that equals 1 if the firm is an environment-friendly manufacturing enterprise.
Control Variables	Corporate Size	Size	The natural logarithm of the total assets.
	Corporate Age	Age	The natural logarithm of the firm’s age.
	State-owned Enterprises	SOE	A dummy variable that equals 1 if the firm is stated-owned and 0 otherwise.
	Financial Performance	ROS	The net profit divided by the operating revenue.
	Financial Leverage	Leverage	The asset-liability ratio.
	Growth Ability	Growth	The growth rate of the operating revenue in the year.
	Tangibility of Assets	PPE	The fixed assets divided by the total assets.
	Investment Level	Invest	The ratio of the investment expenditure to the total assets.
	Cash Holding	Cash	The cash amount divided by the total assets.
	Agency Cost	AgntCost	The sum of the selling expenses and administrative expenses, divided by the total assets.
	Chairman’s Power	Power	A dummy variable that equals 1 if the chairman is also the general manager and 0 otherwise.
	The proportion of Independent Directors	Indpdt	The proportion of independent directors among the board members.
	Shareholding of Senior Executives	MagHold	The shareholding ratio of senior executives.
	Shareholding Concentration	Top	The shareholding ratio of the largest shareholder.
	Equity-control Separation	Separate	The percentage of equity and control separation.
	New Acquired Equity Funds	Equity	The ratio of the newly acquired equity financing amount to the total assets in the current year.
	New Acquired Bond Funds	Bond	The proportion of the newly acquired bond financing amount to the total debt in the current year.
	Regional Fix Effect	Province	A series of dummy variables that represent the provinces.
	Industry Fix Effect	Industry	A series of dummy variables that representing the industries (two-digit industry code).
	Time Fix Effect	Year	A series of dummy variables that representing the years.
